# Eph-ephrin signaling in nervous system development

**DOI:** 10.12688/f1000research.7417.1

**Published:** 2016-03-30

**Authors:** Karina S. Cramer, Ilona J. Miko

**Affiliations:** 1 Department of Neurobiology and Behavior, University of California, Irvine, Irvine, CA, USA

**Keywords:** Ephrins, Eph receptors, neuronal migration, nervous system development, Axon guidance

## Abstract

Ephrins and Eph receptors enable contact-mediated interactions between cells at every stage of nervous system development. In spite of their broad binding affinities, Eph proteins facilitate specificity in neuronal migration and axon targeting. This review focuses on recent studies that demonstrate how these proteins interact with each other, and with other signaling pathways, to guide specificity in a diverse set of developmental processes.

## Introduction

The complexity of nervous system function reflects a vast underlying diversity of neuronal cells and their integration in precise circuitry. During development, newly born neurons migrate to their final destination, become the right type of cell, and form precise connections with their synaptic partners. Given the relatively small number of genes in our genome, how is this complexity generated? A major contributor to the formation of neural circuitry is the Eph family of proteins, which comprises Eph receptors and their ephrin binding partners. These membrane-associated proteins participate throughout neuronal development, during which they display promiscuous binding properties yet specify uniquely targeted events from proliferation to synaptogenesis.

In this large family of signaling molecules, such event specificity does not generally arise from binding selectivity between Eph receptors and ephrins. Instead, multiple modes of Eph-ephrin signaling provide combinatorial codes that differentiate between groups of cells and coordinate multiple aspects of neural development, including cell migration and axon targeting.

Recent studies have demonstrated that differential responses depending on ephrin expression levels and expression of ephrin-associated molecules allow cells born in one place at the same time to adopt distinct migratory routes. In addition, studies have shown how multiple signaling pathways enable distinct axons that grow through a common pathway to terminate in distinct locations.

Specification of neural circuits arises from multiple interacting signaling molecules. The large family of Eph proteins uses both redundancy in expression and multiple modes of signaling to direct this specificity, causing neurons to migrate to their final locations and make synaptic connections there that are appropriate for their function. How can a large family of molecules with broad binding capabilities and redundant expression confer this precision? Here, we discuss recent work focused on factors that determine specificity during cell migration, axon guidance, and midline specializations.

## Eph-ephrin signaling

Eph receptors and ephrins display broad spatial and temporal expression throughout nervous system development. During early development, they contribute to neurogenesis (reviewed in 1) and differentiation ^2^. Some interesting new perspectives that take into account the unique features of Eph-ephrin signaling have emerged.

### Binding within and among cell classes

The Eph receptors are the largest known class of receptor tyrosine kinase. Together with the ephrins, they are divided into the A and B classes on the basis of sequence homology and binding affinities ^3^. The EphA receptors (EphA1-10 in mammals) bind to all ephrin-A molecules (ephrin-A1-6), and the EphB receptors (1–6) bind to all ephrin-Bs (1–3); some variability has been reported in the binding affinities of individual pairs within a class ^4^. There is some crosstalk between classes in that EphB2 can bind to ephrin-A5 and EphA4 can bind to ephrin-B2 ^3, 5, 6^.

### Forward and reverse signaling

Unlike most ligands for receptor tyrosine kinases, the ephrins are associated with cell membranes. Ephrin-B proteins contain a transmembrane domain, and ephrin-A proteins are associated with cell membranes through a glycosylphosphatidylinositol linkage. A major consequence of this membrane association is that Eph-ephrin signaling mediates short-range cell-cell communication, although in some cases soluble forms of ephrin molecules can provide longer-range cues ^7– 9^. The communication between receptors and membrane ligands operates in both directions upon the binding of an Eph receptor in one cell with an ephrin on another cell. Forward signaling refers to signal transduction in a cell expressing the Eph receptor, which upon ligand binding initiates tyrosine phosphorylation. In reverse signaling, signal transduction events are initiated in a cell expressing an ephrin upon its binding with an Eph receptor. Both ephrin-A and ephrin-B proteins can mediate reverse signaling ^10– 12^.

### Attractive and repulsive interactions

In cell migration and axon guidance, movement ultimately relies on the integration of signals that influence the cytoskeleton. Eph-ephrin signaling influences the functions of Rho GTPase proteins, which in turn regulate the actin cytoskeleton (reviewed in 4, 13). Although Eph-ephrin signaling was initially thought to mediate chemorepulsive interactions, later evidence showed that both attractive and repulsive interactions occur. Recent studies have begun to illuminate the factors that determine how Eph-ephrin interactions influence cell migration, adhesion, and axon guidance.

### Combinations and clustering

One potential switch for determining attractive versus repulsive interactions relates to the assembly of higher-order clusters upon ephrin binding to Eph receptors, a prominent feature unique to Eph receptors among the receptor tyrosine kinases ^14, 15^. Cluster formation relies on the ligand-binding domains as well as on interactions between adjacent receptors ^16, 17^. These clusters, needed for initiation of signal transduction pathways, can be expansive and can include more than one type of Eph receptor within a cluster ^18^. Large clusters can include inactive receptors that can be phosphorylated within the cluster; consequently, a single ligand can result in phosphorylation of multiple receptors of both classes ^4, 14, 15^. **

Several new studies have shed light on the role of clustering in the unique responses of Eph-ephrin signaling. EphA4 and EphA2 have similar affinities to ephrin-A5 but have opposing responses in cell assays, whereby EphA4 promotes repulsion while EphA2 promotes adhesion. Seiradake and colleagues ^19^ studied the crystal structure and clustering properties and determined that EphA4 forms small clusters while EphA2 forms large arrays and that these clusters largely determine the cellular response. Along these lines, Klein and colleagues ^20^ prepared clusters of EphB2 of varying sizes and compared effects on phosphorylation, cell collapse, and growth cone collapse. They determined that whilst dimers were associated with a lack of response, trimers and tetramers produced a functional response and that the relative abundance of multimers correlated with the degree of the response. These studies suggest that factors influencing the clustering of Eph receptors are critical for determining how cells will respond to ephrin binding.

### Other interactions in *cis*

Receptor clustering represents a *cis* interaction; that is, two Eph receptor molecules signaling within a single cell. Another type of *cis* interaction observed is the interaction of ephrins and Eph receptors within the same cell, where they are often co-expressed. These interactions have been shown to decrease forward signaling ^21– 23^ and may play an important role in topographic mapping ^24, 25^ as well as axon guidance for spinal motor neurons ^21^.

## Differential control of cortical cell migration

Eph-ephrin interactions regulate cell migration throughout development and are important for establishing boundaries and regulating intermixing of cells ^26, 27^. Several new studies highlight the complexity in the regulation of cell movement by Eph-ephrin signaling. Cell sorting is accomplished by using both repulsive and attractive interactions, both forward and reverse signaling, and a range of selective downstream targets of Eph-ephrin signaling.

### Cerebral cortex development

The diverse and coordinated functions of Eph-ephrin signaling in cell migration are demonstrated by recent studies of cerebral cortex formation. Early in neural tube development, adhesion of neural progenitors to the apical surface is associated with symmetric cell division. A study of null mutations in ephrin-B1 found that these mice exhibited abnormal neuroepithelia and exencephaly ^28^ and that the mutation further disrupted the apical localization of integrin β1. The authors used biochemical assays and culture approaches to show that ephrin-B1 negatively regulates the GTPase Arf6, which is essential for maintaining appropriate integrin β1 localization and adhesion of apical progenitors. In cortical development, some of the first cells to populate the nascent cortical surface are the Cajal-Retzius (CR) cells. This transient population of cells plays a critical role in cortical layering through its release of reelin, which is essential for the characteristic “inside-out” formation of layers. Although apical progenitors require ephrin-B1-mediated adhesion, a recent study used *in vivo* time-lapse imaging together with modeling of cell movement to show that tangential dispersion of CR cells relies on contact-mediated repulsion ^29^. Pharmacological and genetic disruption of Eph signaling showed that both EphA and EphB signaling contribute critically to this repulsion ^29^. Interestingly, the function of reelin in establishing the cortical layers depends extensively on ephrin-B-EphB signaling. Reelin enhances clustering of ephrin-Bs and EphBs, in addition to binding to its receptors. Mutant mice lacking ephrin-B-EphB signaling display severe migration phenotypes with inverted lamination, similar to those seen in *reeler* mice, and activation of ephrin-B-EphB signaling can rescue migration phenotypes in *reeler* mice ^30, 31^.

Another influence of Eph proteins occurs during radial migration. During cortical development, electrical synapses form preferentially between radial glial cells and their sister neurons, forming networks of lineage-related cells in radial columns. When inside-out radial migration is genetically disrupted, the preferential coupling between sister cells is lost ^32^. The tangential dispersion of a subpopulation of these developing cortical neurons promotes crosstalk between clonal columnar units and is significantly reduced in mutant mice lacking ephrin-A signaling ^33^.

### Tangential migration of interneurons

In addition to regulating radial migration and cell dispersion, Eph-ephrin signaling is critical for the tangential migration of interneurons. Excitatory cortical neurons are born at the ventricular zone and migrate radially. In contrast, inhibitory interneurons are generated in the basal telencephalon and migrate extensively along a tangential route. Cortical interneurons born in the medial ganglionic eminence (MGE) migrate along a deep route, whereas preoptic area (POA)-derived interneurons migrate along a superficial route. These routes exhibit complementary expression of EphA4 and ephrin-B3, respectively. A study using organotypic cultures and stripe assays together with gene knockdown and pharmacological approaches showed that forward signaling through EphA4 and reverse signaling through ephrin-B3 induce repulsion of MGE and POA interneurons in the inappropriate routes ^34^. MGE interneurons also express ephrin-As, and reverse signaling induced in ephrin-As by EphA4 enhances motility in these migrating MGE interneurons ^35^.

The POA also generates striatal neurons, which are generated at a similar time and also express ephrin-B3. However, striatal neurons traverse an intermediate route and terminate in the striatum, which expresses EphB1. Reverse signaling through ephrin-B3 elicited by striatal EphB1 is repulsive for migrating cortical interneurons but is an attractive stop signal for migrating striatal cells ^36^. What accounts for these divergent effects? In cortical interneurons, EphB1-ephrin-B3 reverse signaling leads to enhanced phosphorylation of Src tyrosine kinase and of focal adhesion kinase (FAK), leading to enhanced repulsion. For striatal neurons, this same signaling leads to a dephosphorylation of Src and FAK, which leads to attraction ^36^. These divergent effects might arise from endogenously high levels of phosphorylated Src and FAK in striatal cells and/or to distinct combinations of transcription factors in these two cell populations that can influence elements of signal transduction pathways.

Striatal interneurons are generated in the MGE along with cortical interneurons. They are attracted to the striatum through Nrg1/ErbB4 signaling and are simultaneously repelled from the adjacent cortex through EphB forward signaling ^37^. These authors used chromatin immunoprecipitation and luciferase assays to show that the expression of EphB1 and EphB3 is enhanced by Nkx2-1, which is expressed in striatal but not in cortical interneurons born in the MGE.

Eph-ephrin signaling thus provides both repulsive and attractive cues for tangentially migrating neurons. The determination of migratory route depends on the ensemble of Eph receptors expressed along with the molecular context within cells destined for different routes.

## Axon guidance

### Topographic mapping

The function of Eph-ephrin signaling in axon guidance was originally discovered in the context of topographic mapping. Graded expression of ephrin-As in the optic tectum and opposing gradients of EphA receptors were found to be necessary for forming the high degree of topography seen in this pathway ^38– 40^. Since then, further evidence has shown that Eph-ephrin signaling is critical for establishing topographic projections in the auditory system ^10, 41, 42^, somatosensory system ^43^, olfactory system ^44^, and others ^45– 47^. Both forward and reverse signaling play a role, as do attractive and repulsive interactions and interactions in *cis.*

Formation of topography thus uses graded, or continuous, targeting signals. However, Eph-ephrin signaling also contributes significantly to selection of discrete, or discontinuous, synaptic targets, and these modes of targeting often occur together in a single neural pathway. Interestingly, the function of individual Eph or ephrin family members is specialized within a structure, so that family members regulating topography are distinct from those regulating modular ^48^, laminar ^49^, or ipsilateral versus contralateral ^50^ axon targeting decisions.

### Choice points

Eph-ephrin signaling plays an important role in axon targeting at choice points, where axons select between two alternative routes. For example, null mutations in EphB1 result in reduced numbers of retinal ganglion cells that project ipsilaterally through the optic chiasm to the ipsilateral region of the lateral geniculate nucleus ^51^. This phenotype is recapitulated when the cytoplasmic domain of EphB1 is deleted, suggesting that reverse signaling is not necessary for this targeting choice. Conversely, overexpression of EphB1 in mouse embryos is sufficient to direct retinal ganglion cell axons to the ipsilateral trajectory ^52^. Interestingly, loss or gain of EphB1 reduces or increases ipsilateral targeting, respectively, whereas changes in EphB2 and EphB3 are relatively ineffective, even though these receptors are co-expressed to varying degrees in retinal ganglion cell axons ^51, 52^. Analysis of the effectiveness of overexpressed chimeric receptors suggests that unique sequences in both the juxtamembrane and the extracellular domains of EphB1 work together to direct axons ipsilaterally. The selective role for EphB1 might result from differences in its ability to engage downstream signaling pathways. Additionally, crossing axons might express additional proteins that normally overcome this ipsilateral cue. In this study, the authors overexpressed the zinc finger transcription factor *Zic2*, which is expressed in retinal ganglion cells and which activates EphB1 and regulates numerous other genes as well. They found that early exogenous expression of *Zic2* was significantly more effective at inducing ipsilateral projections than EphB1, consistent with the view that a network of genes is needed to balance responses to ipsilateral versus contralateral cues. The identification of these genes and the integration of their roles in target selection, which may require a computational modeling approach, will greatly facilitate our understanding of how Eph-ephrin signaling leads to precision in axon targeting.

Several other studies highlight the broad significance for Eph-ephrin signaling in determining whether axons cross the midline ^53– 56^. Eph family molecules play key roles in establishing the crossed projections of the central nervous system ^54, 57^. Repulsion from ephrins expressed at the midline may serve to limit crossing projections spatially ^58^ or temporally ^53^. Eph-ephrin signaling is a significant factor in determining whether axons make ipsilateral or contralateral synaptic target selections. This role has been demonstrated in the auditory brainstem pathway from the cochlear nucleus to the medial nucleus of the trapezoid body, a strictly contralateral projection in the normal brain. Mutations that reduce reverse signaling through ephrin-B proteins ^59^ and null mutations in ephrin-A2 or ephrin-A5 (or both) similarly reduce the specificity of this pathway, resulting in a significant ipsilateral projection ^50^. The similarity in these phenotypes suggests crosstalk between the classes and redundancy in cues for generating the crossed projection. Unlike the optic chiasm, in which a subset of axons is selectively targeted ipsilaterally by EphB1, this auditory projection uses multiple Eph-ephrin signaling molecules to prevent the formation of *any* ipsilateral projections. In this case, downstream signaling molecules might be similarly engaged by both classes of Eph proteins.

Recent studies of motor neuron axon guidance have shed new light on the molecular mechanisms by which Eph-ephrin signaling coordinates distinct choices. Two groups of motor neurons of the lateral motor column (LMC) in the spinal cord innervate the limb. The medial LMC (LMC _M_) motor neurons innervate ventral limb muscles, whereas the lateral LMC (LMC _L_) motor neurons innervate the dorsal limb muscles ^60^. The axons of the LMC motor neurons grow out of the spinal cord together in one fascicle and make a dorsal versus ventral choice as they enter the limb. Another group of motor neurons in the medial portion of the medial motor column (MMC _M_) at the level of the hindlimb initially project axons together with the LMC axons but abruptly change course toward dorsal axial muscle targets. Both LMC _L_ and MMC _M_ axons express EphA4 and encounter ephrin-A5 along their trajectories. *In ovo* electroporation studies in chick embryos revealed that the two populations have opposite responses to ephrin-A5: LMC _L_ axons avoid ephrin-A5 in the limb, whereas MMC _M_ axons grow through ephrin-A5-positive somite regions ^61^.

The distinct responses of these EphA4-positive axons are further complicated by the fact that these axons also express ephrin-A proteins. EphAs and ephrin-As in LMC _L_ motor axons are maintained in separate membrane compartments ^62^, so that *trans* interactions are favored, whereas in LMC _M_ motor axons, EphAs and ephrin-As can reside in the same membrane compartments and interact in *cis* ^21^. LMC _M_ motor neurons are also guided by EphB signaling ^63^.

In the *trans* signaling guiding LMC _L_ motor neurons, the effects of forward signaling through EphA receptors are repulsive, whereas reverse ephrin-A signaling is attractive ^62, 64^. Both forward and reverse signaling are necessary to target LMC _L_ axons to the dorsal limb. Using co-immunoprecipitation assays to identify novel ephrin-A co-receptors, Bonanomi and colleagues ^64^ found that the selective attraction of LMC _L_ axons through ephrin-A reverse signaling is mediated by Ret, a tyrosine kinase. Ret also interacts with GFRα1, a GPI-linked receptor for glial-derived neurotrophic factor, which is secreted in the limb. Ret thus integrates signals from these two sources and generates a synergistic interaction that promotes axon attraction. This study highlights the significance of combinatorial codes in establishing diversity and precision in neuronal contacts.

## Midline specializations

### Corpus callosum

Eph proteins play a key role in establishing midline structures. In humans, mutations in *EFNB1*, the gene coding for ephrin-B1, leads to craniofrontonasal syndrome (CFNS). This syndrome is characterized by abnormally large distance between the eyes, a central nasal groove, cleft palate, and skeletal/sternum abnormalities, along with other midline distortions in the body. CFNS is also associated with agenesis of the corpus callosum, a large neural tract that interconnects the cerebral hemispheres ^65, 66^.

The mouse model for CFNS parallels many of the cranial deformities and also exhibits incomplete formation of the corpus callosum ^65^, which depends on ephrin-B1 reverse signaling ^67^. Deeper examination of corpus callosum formation in several Eph family mutant mice revealed axon outgrowth defects near the midline, after axons have progressed out of cortical layers and traveled medially toward their contralateral journey. These axons coalesce and turn to project longitudinally, not medially via the commissure, similar to the human CFNS phenotype ^54^. Axon guidance across the midline is largely dependent on ephrin-Bs and EphBs, which are expressed in growing callosal fibers. Furthermore, abnormal glial proliferation at the midline in mutant mice brains suggested that Eph family proteins regulate this population through growth suppression ^54^. These studies suggest that agenesis of the corpus callosum in CFNS results from defects in axon guidance regulated by Eph-ephrin signaling.

### Neural crest

Owing to the large morphological impact of the *EFNB1* mutation in CFNS, investigations into the mechanisms have focused on earlier developmental stages. Indeed, *in vivo* manipulation of ephrin-B1 expression in the developing mouse results in perturbations of cephalic neural crest cell precursors ^68^, which give rise to the bone and cartilage of the head. Whereas neural crest cell migration is known to be regulated by Eph proteins ^69^, craniofacial defects in ephrin-B1 mutants largely arise from impaired cell proliferation in the developing palate ^68^ as well as from impaired cell survival ^70^. Craniofacial development relies on ephrin-B1 reverse signaling ^67^. Interestingly, loss of ephrin-B1 in the developing palate leads to increased expression of EphB3, as forward signaling normally modifies EphB3 so as to promote endocytosis and degradation ^68^. Together, these studies show that loss of ephrin-B1 affects several aspects of central and peripheral development through multiple molecular interactions.

## New research directions

Apart from CFNS, an understanding of the impact of mutations affecting Eph-ephrin signaling on human brain conditions is in its very early stages. Genetic studies have linked these mutations with neurodevelopmental disorders, including autism spectrum disorder (ASD) ^71^, characterized by dysfunction in social interactions, repetitive behaviors, and sensory abnormalities. Some of these behaviors can be identified in simplified form in mouse models of ASD. To explore the potential link with Eph proteins, Wurzman and colleagues ^72^ performed a comprehensive series of behavioral tests on ephrin-A2/A3 double-knockout mice. In a three-chamber social interaction test, the knockout mice spent significantly less time than wild-types did in a chamber exposed to a novel mouse, indicating social aversion. Compared with wild-type mice, the ephrin-A2/A3 mice exhibited significantly greater repetitive and self-injurious grooming behavior. They also showed decreased acoustic startle response and increased prepulse inhibition of the startle reflex. These behavioral phenotypes are similar to those in other mouse models of ASD. This work, though still in its early stages, expands the relevance of developmental Eph-ephrin signaling in establishing normal sensory function and behavior.

## Summary

A large body of work on the roles of Eph proteins and the mechanisms underlying their versatility has emerged. Recent work demonstrates the breadth of roles throughout development and the significance for assembly of sensory, motor, and cognitive neural systems. Determining how these multiple functions are coordinated remains a significant challenge. The recent studies highlighted here have begun to shed light on this complex issue, showing that specificity arises from differential clustering, forward and reverse signaling, and unique combinations of protein family members that engage distinct signaling pathways.

## Abbreviations

ASD, autism spectrum disorder; CFNS, craniofrontonasal syndrome; CR, Cajal-Retzius; FAK, focal adhesion kinase; LMC, lateral motor column; LMC _L_, lateral lateral motor column; LMC _M_, medial lateral motor column; MGE, medial ganglionic eminence; MMC _M_, medial motor column; POA, preoptic area.
